# An Update on the Management of Rectal Neuroendocrine Neoplasms

**DOI:** 10.1007/s11864-024-01267-4

**Published:** 2024-10-30

**Authors:** Aviva Frydman, Raj Srirajaskanthan

**Affiliations:** 1 Neuroendocrine Tumour Unit, King’s College Hospital, London, UK; 2 Department of Gastroenterology, Kings College Hospital, 2Nd Floor Hambleden Wing, Denmark Hill, London, SE5 9RS UK

**Keywords:** Rectal neuroendocrine tumours, Endoscopic ultrasound, Endoscopic resection, Lymphovascular invasion, Systemic therapies

## Abstract

Rectal neuroendocrine neoplasms (NENs) are increasing in incidence. Most lesions are low grade, well-differentiated neuroendocrine tumours with good long term outcomes. However there is metastatic potential and resection offers the only option for a cure and in most cases should be offered to reduce the risk of metastases. Careful staging of rectal NENs should be performed prior to consideration of resection in order to ensure the appropriate technique is chosen, and reduce the risk of incomplete resection. Resection can be endoscopic or surgical, and selecting the appropriate resection technique relies on tumour characteristics such as size, grade, invasion into the muscularis propria, presence of lymph node involvement or of distal metastases. Some patients may require systemic therapies which may involve somatostatin analogues (SSAs), everolimus, tyrosine kinase inhibitors (TKIs), chemotherapy or peptide receptor radionuclide therapy (PRRT). Due the rarity of these tumours, much of the evidence is based on retrospective reviews or smaller cohort studies. This article is an update of the current evidence available to guide management.

## Introduction

There has been a reported increase in the incidence of rectal neuroendocrine neoplasms (NENs), likely in part due to an increase in colonoscopies performed for bowel cancer screening [1, 2]. However, some of this increase in incidence is being driven by new diagnoses in younger people below 55 years of age who are not typically screened for bowel cancer [3]. This may be related to increased use of colonoscopy for investigation of gastrointestinal symptoms in younger patients. Neuroendocrine neoplasms can be differentiated into well-differentiated neuroendocrine tumours (NETs) and poorly-differentiated neuroendocrine carcinoma (NEC) [4]. Most rectal NENs are diagnosed as small well-differentiated NETs, which generally have good long-term survival. Despite this, multidisciplinary management is essential to reduce the risk of metastatic disease and improve long term outcomes.

There is global variation in the incidence of rectal NETs, with a preponderance of these lesions identified in Asian countries where rectal NETs account for 29.6 to 50% of all gastroenteropancreatic (GEP)-NET [5–8]. Hence, a significant proportion of the published data is from this region of the world. The SEER database also suggests different incidence of rectal NETs in ethnic groups with highest rates in Hispanic, Black and Asian populations [9]. The high incidence in Asian groups suggests that genetic rather environmental factors may be contributory.

Over 80% of rectal NETs are < 10 mm and low grade at diagnosis [5]. This data is from large multi-centre studies predominantly from Asia. It is unclear as to whether the rectal NETs seen in Western counties have the same size distribution. However most lesions were < 10 mm in the largest European series [10]. Small rectal NETs < 10 mm have a low risk of metastases, however all rectal NETs have metastatic potential and therefore should be offered resection. The optimal method depends on patient and tumour characteristics which should be determined before resection, with the aim of reducing the risk of incomplete resection. Unfortunately, this is not always the case as rectal NETs are commonly not recognized at the time of screening colonoscopy. Incomplete resection is associated with poorer outcomes, therefore determining the optimal approach is a critical step in the management of rectal NETs [2].

The European Neuroendocrine Tumor Society (ENETS) released updated guidelines for the management of rectal NETs in 2023 [11], however management questions remain. Notably, these guidelines combined colonic and rectal neuroendocrine neoplasms which may require different approaches. A key remaining question is which tumours can be resected endoscopically, and which endoscopic technique should be used. Given the relatively low incidence of these tumours, much of the evidence consists of single-centre or retrospective studies.

This review discusses an update in the resection options, how to select the appropriate management as well as potential systemic therapies for those with residual or recurrent disease.

### Preoperative Investigations

**Fig. 1:**
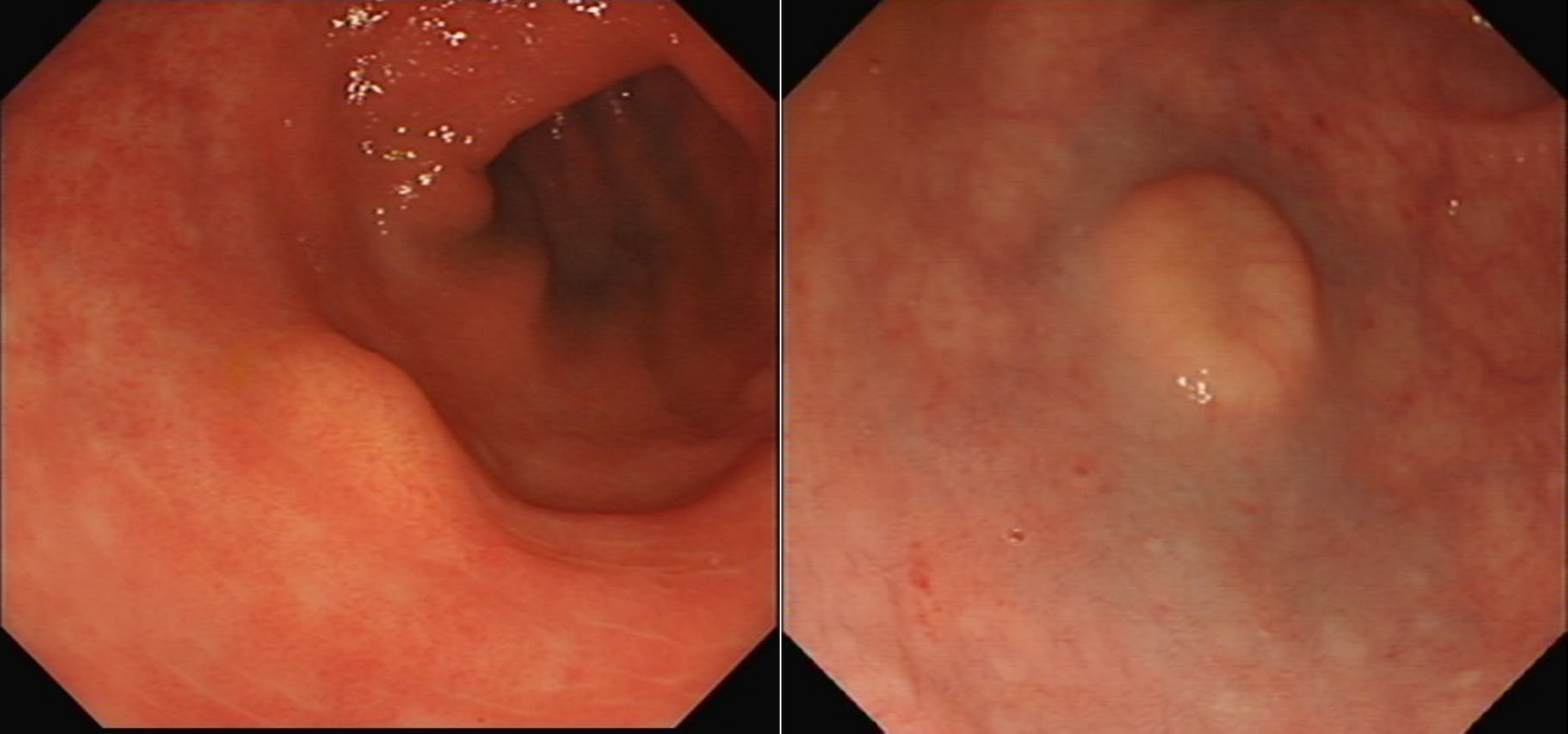
Endoscopic imaging of rectal NETs showing the mucosal appearance on the left, and central depression on the right

Some rectal NETs may be diagnosed as a result of symptoms, however many are diagnosed incidentally at colonoscopy [2]. This may result in snare polypectomy which carries a high risk of incomplete resection and residual disease [12]. A key factor in appropriate management is in recognizing lesions as NETs at the time of colonoscopy. Endoscopic characteristics of NET may include a yellow/orange colour, irregular surfaces, a doughnut-shaped lesion with central depression, particularly for lesions > 10 mm [11, 13]. Central depression (Fig. 1) may be a sign of lymph node involvement, and therefore its especially important that pre-operative tumour staging occurs to reduce the risk of incomplete resection. Lesion identification and characterization is an important area for education of endoscopists. There are multiple online resources for example UEGW tool (https://ueg.eu/p/97) to help endoscopists characterize these lesions. A French multi-centre study noted that only 18% of rectal NETs were diagnosed / suspected at index procedure. This led to 76% having incomplete resection at first procedure [10].

Once a lesion is recognized as being a NET, either by endoscopic characteristics or by biopsy proven histopathology, selecting the correct resection technique then depends on tumour characteristics. Guidelines recommend that resection of rectal NETs depends on tumour size, grade and presence of lymph node involvement [11]. Lesion size is a key factor in the risk of lymph node involvement and of metastasis, and rectal NETs ≤ 10 mm are unlikely to have lymph node involvement (1–10%) [11]. For rectal NETs ≤ 10 mm guidelines recommend endoscopic resection, and lesions > 20 mm are recommended to under oncological resection. For lesions 10 – 20 mm, the appropriate surgical technique is uncertain and depends on tumour characteristics which should be determined by the preoperative investigations and multidisciplinary discussion [11, 13].

When deciding resection technique, it is important to balance the potential risks of treatment against the risk of incomplete resection. Oncological resection is not recommended for small tumours < 10 mm with no evidence of lymph node involvement due to the low risk of metastatic disease and the high morbidity associated with surgery [14]. However, it is important to recognize that there remains a risk of metastases even in small rectal NETs ≤ 10 mm, and the risk increases with increasing tumour size. A study of 132 patients with rectal NETs showed that there were 0% lymph node metastases in tumours ≤ 6 mm, but 10.3% in tumours sized 7 – 10 mm [15]. Another retrospective study of 32 patients reported a high percentage of lymph node involvement of 96.9% in patients with lesions ≤ 20 mm [16]. Another small study demonstrated 25% node involvement in tumours ≤ 10 mm [17]. Therefore, imaging is required to determine the degree of invasion as this should determine the approach to resection.

Endoscopic ultrasound (EUS) assists with judging the size of the lesion, depth of invasion and presence of lymph node metastasis. Recent ENETS guidelines do not specify when EUS should be used [11]. An Italian position paper recommend performing EUS for lesions > 10 mm before resection [13]. We perform EUS for all lesions > 5 mm to ensure there is no invasion into the muscularis propria prior to resection [2]. If there is muscular involvement and no lymph node involvement, endoscopic submucosal dissection (ESD) may be preferred over endoscopic mucosal resection (EMR). Lesions smaller than 5 mm may not be visible.

**Fig. 2:**
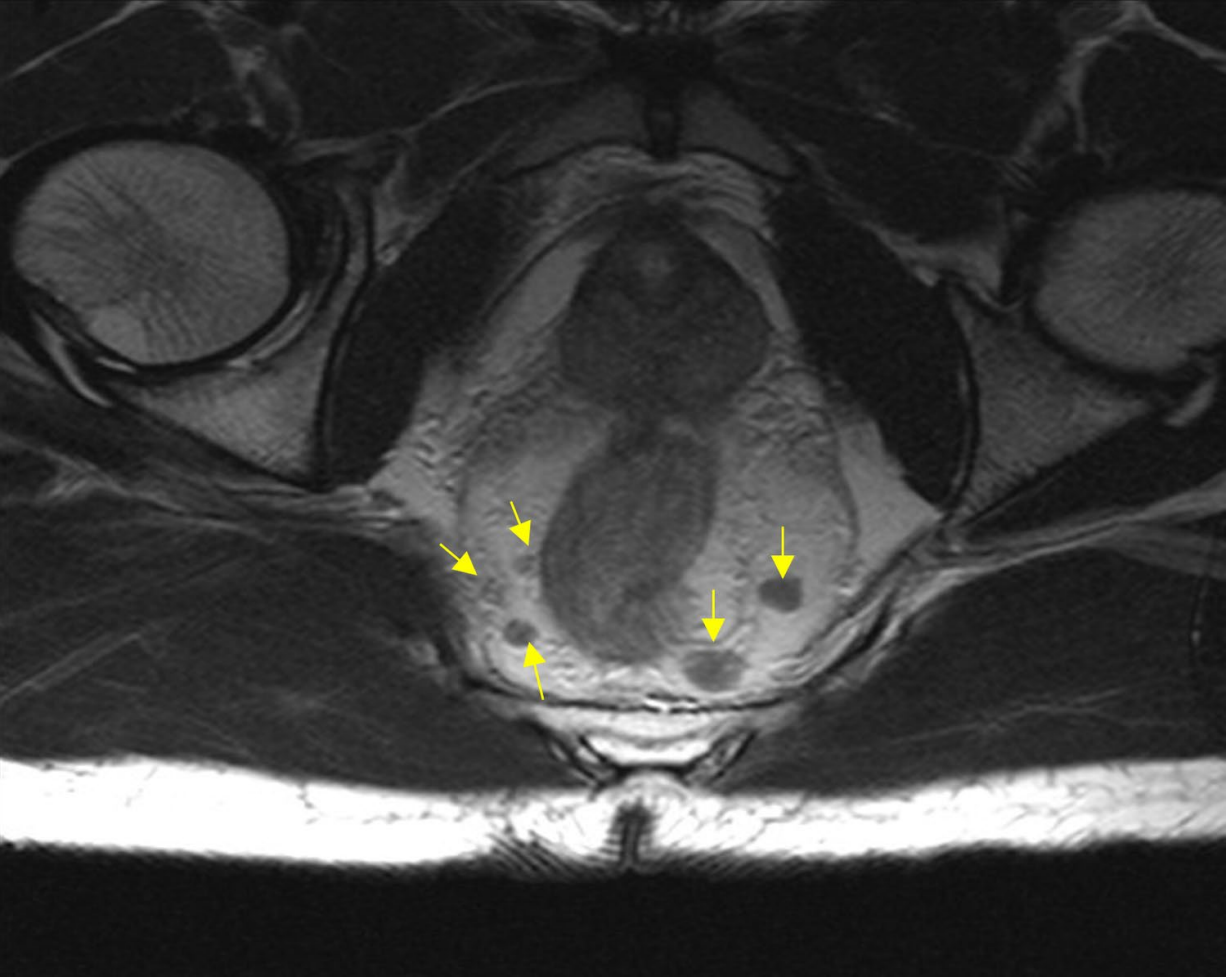
Staging MRI scan which shows multiple lymph node metastases as denoted by arrows

Magnetic resonance imaging (MRI) helps to identify lymph node metastases, which is particularly important in tumours larger than 10 mm (Fig. 2). ENETS recommend all tumours ≥ 10 mm, all G2-G3 tumours and all with suspected lymph node involvement on EUS should undergo MRI prior to surgical resection [11]. In our practice all lesions ≥ 5 mm undergo formal staging with MRI [2]. This is in part due to low accuracy of sizing lesions at time of endoscopy. If there is any concerning lymph node involvement on MRI, functional imaging should be undertaken with Ga-68 DOTATATE PET as well as a staging CT chest, abdomen and pelvis scan.

Grade of tumour is also important as G2 and G3 rectal NETs have a higher risk of metastatic spread and grade is an independent risk factor for recurrence [2]. Therefore, if the diagnosis is made from biopsy that suggests a grade 2 or 3 lesion, these patients should have a baseline MRI scan prior to resection and if there is nodal disease present, further imaging with a Ga-68 DOTATATE PET or staging CT scan should be considered. Furthermore, EUS is important to ensure there is no deep muscle involvement which would alter the therapeutic approach.

### Primary Resection

#### Endoscopic Resection

Endoscopic resection is the standard approach for lesions < 10 mm, and may be considered in lesions 10 – 20 mm depending on tumour characteristics [11]. Endoscopic mucosal resection (EMR) and endoscopic submucosal dissection (ESD) are common methods for rectal NETs ≤ 10 mm. Other endoscopic methods include endoscopic full thickness resection (EFTR) and transanal endoscopic microsurgery (TEMS). There is conflicting evidence as to which modality is preferred and most data comes from single-centre or retrospective studies. Many studies occurred in Asia where endoscopic proficiency may be higher. This is highlighted when comparing the reported 71.4–100% R0 resection rates achieved in an Asian series with lesions < 20 mm [18] compared with that achieved in a French series of 39% R0 resection [10].

Previous studies have shown that standard EMR is inferior to ESD as it is less likely to achieve full resection due to the high rate of positive vertical margins, however modified EMR techniques have been developed, which have been reported to be equivalent or superior compared to standard EMR [19]. Various modified techniques exist including cap-assisted EMR, underwater EMR and EMR with a ligation device (EMR-L) [20].

A single-center randomized trial of 79 patients with rectal NETs ≤ 10 mm demonstrated that modified cap-assisted EMR was non-inferior to ESD in terms of rate of complete resection [21]. As cap-assisted EMR was a shorter procedure with a no significant difference in complications, it was considered to be favourable. It is noted that patients with nodal involvement or distant metastases were excluded.

A multi-centre retrospective study demonstrated that there was no significant difference in complete resection rate for EMR-L compared to ESD [22]. In EMR-L, a ligation device allows the lesion to be sucked in to the cap of the scope and a rubber band fired over the lesion prior to performing a snare resection. This aids achieving an R0 resection. In this study EUS was performed to rule out muscle layer involvement and the median tumour size was 5 mm which is consistent with evidence that in smaller tumours without deep invasion EMR is equivalent to ESD, however this may not be the case with larger tumours or those with muscle layer involvement. The study protocol for a prospective multicenter RCT comparing EMR-L to ESD has been recently published which aims to recruit 266 patients across multiple institutions in Japan [23].

A systematic review and meta-analysis of 18 retrospective studies including 1168 patients found that for rectal NETs < 10 mm, there was no statistically significant difference in complete resection or complication rates between EMR and ESD groups [20]. This analysis included both conventional and modified EMR techniques. Another meta-analysis of 14 studies including 813 patients revealed that for lesions < 10 mm EMR with suction had a higher complete resection rate than ESD [24].

Another recent prospective study of 79 patients demonstrated that for lesions < 10 mm, EMR and ESD both achieved complete resection in all patients [18]. However for lesions between 10 to 20 mm, ESD achieved 100% complete resection, and EMR only 71.4%. They conclude that for lesions < 10 mm, EMR is preferred as it is a shorter procedure, whereas for lesions 10 – 20 mm, ESD is preferred due to the higher complete resection rate. A limitation of this study is that it compared standard rather than modified EMR.

There are limitations of the current available data. Many studies occurred at single centres, and therefore findings may be subject to specific differences in endoscopic skills and training between sites favoring one technique over the other. Retrospective studies often occurred during the period that new EMR techniques were being developed, therefore may underestimate modified EMR success rates. The meta-analyses have included studies primarily performed in Asian countries and hence there may be bias due to endoscopic expertise.

Overall, for lesions ≤ 10 mm, EMR and ESD appear to be comparable. However, if there is evidence of muscularis propria involvement, even for lesions ≤ 10 mm, ESD or surgical resection may be preferred. In our practice, we generally perform ESD for all suitable rectal NETs under 10 mm, since this enables the operator to be sure the dissection under the lesion is complete and we can achieve an R0 resection in the deep margin. Table 1 summarizes the recommendations for resection based on tumour size.

**Table 1:** Summary of resection techniques and recommendations based on tumour size

Lesion size	Resection techniques	Advantages and disadvantages	Recommendation
< 10 mm	Traditional-EMR	Fast procedure, higher rates incomplete resection compared to ESD	Not recommended
	Modified-EMR	Fast procedure, modified technique depends on local expertise, high rates complete resection	Consider if no muscle layer involvement and grade 1 lesions
	ESD	Longer procedure, depends on experienced endoscopist, high rates complete resection	Preferred if muscle layer involvement
	EFTR	High rate of complete resection, minimal post-operative complications, shorter learning curve. Difficulty ensuring clear margins	Consider for resection if no ESD or modified EMR expertise
10–20 mm	ESD	Lower rate of complete resection depending on expertise	Suitable in selected cases
	TEMS	High rate of complete resection, risk of post-operative faecal incontinence	Suitable for this size of lesion
	EFTR	Higher rate of complete resection, minimal post-operative complications, difficulty with lateral margins for larger lesions due to size of cap	Can be considered
> 20 mm or evidence of lymph node involvement	Oncological resection	Best chance of complete resection	Recommended

EMR: endoscopic mucosal resection; ESD: endoscopic submucosal dissection; EFTR: endoscopic full thickness resection; TEMS: transanal microsurgery

Choosing the resection approach for lesions size 10 – 20 mm is difficult due to limited evidence. Current guidelines recommend full imaging and MDT discussion to determine the appropriate technique. Transanal microsurgery (TEMS) is a localized resection approach which confers the ability to completely remove the lesion, however there is a risk of postoperative fecal incontinence [14] due to prolonged dilatation of the anal canal during procedure. A recent systematic review and meta-analysis showed that for tumours ≤ 20 mm, TEMS had a higher rate of complete resection compared with ESD [25].

Endoscopic full thickness resection (EFTR) was developed to enable endoscopic resection of lesions involving or arising from the muscle layer. Lesions are drawn into the cap of the endoscope either by suction and/or grasping forceps, a large clip is deployed over the lesion and closing the mucosal layers beyond this point. Then a snare resection is performed and this cuts through the entire bowel wall including serosa, enabling a full thickness resection. The benefit is that complete resection can be achieved, the device is easier to use and the learning curve is faster than ESD. Reported complications include perforation due to failure of clip deployment, fistualisation, post-polypectomy syndrome, abdominal pain. A retrospective multi-centre German study of 40 patients who underwent EFTR had complete resection in 100% of cases, of which 27 patients had tumours < 10 mm, and 12 patients had tumours between 10–20 mm [26]. There were no major adverse events.

A recent large retrospective review of 453 patients who underwent resection included 60 patients with lesions measuring 10–20 mm in size, making up 13.2% of the cohort [27]. Of this group, 22 of them had radical resection, the others had endoscopic resection. They found that size and grade were predictive factors for lymph node metastasis in tumours 10 – 20 mm in size. They suggest that for patients with tumours 10 – 20 mm in size with grade 2 or 3 tumours, surgical resection should be considered rather than endoscopic. Limitations are the small number of patients included with tumour size 10—20 mm, single-centre and the nature of retrospective study. Outcomes for endoscopic resection compared to surgical resection was not reported.

#### Oncological Resection

Lesions ≥ 20 mm or those with evidence of lymph node metastases should undergo oncological surgical resection with either anterior resection (AR) or abdominoperitoneal resection (APR) with total mesorectal excision (TME), as the likelihood of lymph node and distant metastases is higher [11]. This requires careful counselling of patients as there is potential for significant morbidity.

#### Watch-and-Wait

There may be a potential role for watch-and-wait as per case reports demonstrating no growth after prolonged surveillance [28]. However, a large retrospective review of 16,531 patients on the United States National Cancer Database demonstrated that when compared to local excision, observation alone was associated with a poorer 5-year overall survival [29]. Note that in this review, only a small percentage of the patient population did not undergo surgery (12.4%). We recommend that watch-and-wait strategy only be adopted where resection is contraindicated or due to patient preference after appropriate counselling.

#### Incomplete R1 Endoscopic Resection

Rectal NETs are often found to be neuroendocrine in nature after a routine polypectomy, and hence are often incompletely resected. To determine whether to repeat resection, investigations should be performed to determine the risk of disease progression or recurrence. Patients should undergo MRI and EUS evaluation of the scar area prior to second resection. The EUS is important to assess the remaining submucosal tissues and the presence of lymph node involvement. A biopsy of the scar may be useful even if there is no evidence of disease recurrence on endoscopy and ultrasound, as there may still be residual disease that was not visible [30]. If there is evidence of residual disease in small tumours without lymph node involvement, ESD is often required to achieve complete resection [2]. The published data on incomplete resection is very limited due to the high rates of R0 resection achieved in Asian series. Data from European multi-centre studies demonstrate higher rates of R1 resection and in Western practice incomplete resection is a common problem [10]. The most common causes are resection technique and size of the tumour. Incomplete resection can be categorized as R1 microscopic involvement of margins or R2 when visible tumour is present following resection.

The significance of an incomplete resection is still unknown, since many of these lesions are small and in published data on recurrence or metastatic disease in rectal NETs, incomplete resection does not appear to be a factor. However, this may be partly due to limited data available. The benefit of an R0 resection in grade 1 lesions < 10 mm is that the known risk of recurrence and development of metastases is very low and therefore patients can be discharged. However, for all grade 2 or 3 cases and lesions > 10 mm there are recommendations for follow up due to risk of developing metastatic disease, even following an R0 resection.

Size and grade of the lesion should be considered when deciding on repeat resection technique. Grade 1 lesions < 10 mm without lymphovascular invasion who have no residual disease on scar biopsy do not require a salvage procedure. However, there may be benefit from repeat resection of scar, because if an R0 resection is achieved then patients can be discharged from follow up if there are no high risk factors. A recent multi-centre study from France demonstrated residual disease in 43% of scars resected for patients with a previous R1 resection [31]. Of these, 75.7% achieved R0 resection after second endoscopic procedure, who were then able to be discharged from further surveillance.

**Fig. 3:**
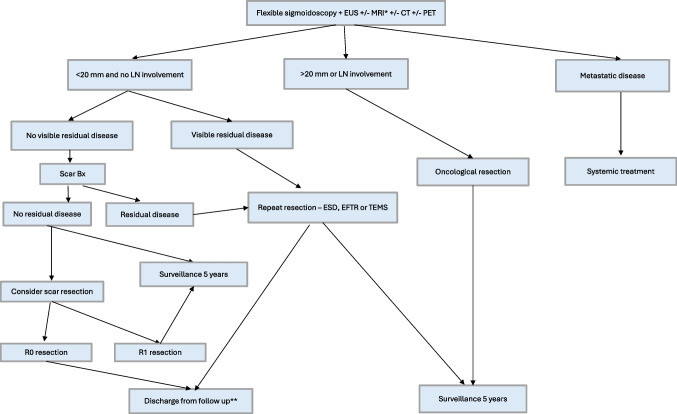
Suggested approach to incomplete endoscopic resection. *MRI if lesion > 10 mm or G2/G3 histology. CT and/or PET to be considered if evidence of nodal disease on MRI scan. **If R0 achieved and total lesion size < 10 mm and grade 1 consider discharge from follow up

For lesions < 10 mm that are grade 2 or 3 with no residual disease on endoscopy, ESD can be considered. For those with visible residual disease, a salvage resection should be performed with the goal to achieve complete resection. Patients with lesions > 20 mm where an endoscopic resection has been performed without clear margins should undergo oncological resection. When considering repeat / salvage procedures to remove residual disease, there is no role for EMR techniques, instead ESD, EFTR or TEMS should be considered as long as there is no concern for loco-regional or metastatic disease. A proposed algorithm for management of incompletely resected NETs is described in Fig. 3.

#### Surveillance/ Follow up Recommendations

Grade 1 lesions ≤ 10 mm with complete resection and no lymphovascular invasion are considered cured and do not require follow up [11]. Incomplete resections of grade 1 lesions ≤ 10 mm that do not go on to have complete resection should have 12 monthly MRI pelvis and sigmoidoscopy. In grade 1 lesions ≤ 10 mm with lymphovascular invasion, follow up with MRI and sigmoidoscopy should be done for 5 years, as well as an initial Ga-68 DOTATATE PET, and repeated at 12 months..

Grade 2 lesions ≤ 10 mm should have 6 monthly MRIs and annual sigmoidoscopy for 5 years. For these lesions an initial Ga-68 DOTATATE PET should be done and repeated at 12 months. The same applies to any lesion > 10 mm which has been completely resected.

All grade 3 lesions which have been completely resected should have MRI every 3 months for 2 years followed by 6 monthly MRI for a further 3 years, as well as 6 monthly sigmoidoscopy for 2 years, then annual endoscopy for a further 3 years. They should have an initial Ga-68 DOTATATE PET and repeat at 12 months (See Table 2).

**Table 2:** Follow-up advice given at our institution based on grade, size of lesion and type of resection

Grade	Size	LVN*	Type of resection	Follow-up advice
G1	< 10 mm	Negative	R0	Discharge
	< 10 mm	Negative	R1	Consider repeat resection, if R0 can be discharged [31]
	< 10 mm	Positive	R0	MRI every 6 months and flexible sigmoidoscopy every 12 months for 5 years Initial Ga-DOTATATE-PET and repeat at 12 months
G2	< 20 mm		R0	MRI every 6 months and sigmoidoscopy every 12 months for 5 years Initial Ga-DOTATATE-PET and repeat at 12 months
	< 20 mm		R1	Repeat resection, followed by 6 monthly MRI and sigmoidoscopy every 12 months
	> 20 mm		R0 or R1	Oncological resection followed by 6 monthly MRI for 5 years
G3	Any		R0 or R1	MRI every 3 months for 2 years, then every 6 months for a further 3 years 6 monthly sigmoidoscopy for 2 years then annual for a further 3 years Initial Ga-DOTATATE-PET and repeat at 12 months. Consider FDG-PET at baseline

*Lymphovascular or nodal involvement

#### Systemic Therapies

The majority of rectal NENs are small with localized disease. A small percentage can present with locoregional or metastatic disease, and some patients may develop metastatic disease recurrence. Rectal NETs therapies are discussed in the section below. Rarely, rectal NECs can occur and treatment for these lesions are usually platinum based chemotherapy agents such as carboplatin and etoposide.

#### Somatostatin Analogues

Somatostatin analogues (SSAs) are standard first-line treatments in metastatic or unresectable NETs, however there is no published data specifically investigating their use in rectal NETs. Small numbers of patients with hindgut tumours were included in the CLARINET trial and open-label extension study which showed prolonged progression-free survival (PFS) in lanreotide group compared to placebo [32, 33]. There was no subgroup analysis for outcomes in rectal NETs. The PROMID study of octreotide included only midgut tumours, therefore excluded patients with rectal NETs [34]. A recent study showed that 70% of rectal NETs were somatostatin receptor 2 positive, and positivity was associated with better overall survival [35]. Given that SSAs target somatostatin receptors on the surface of NET cells, this may suggest that SSAs could be effective for the majority of rectal NETs. ENETS recommend they may be used in tumours that are somatostatin receptor positive and slowly growing [11].

#### Everolimus

The mTOR inhibitor everolimus has been demonstrated to be effective as an anti-proliferative agent in patients with gastrointestinal NETs in the RADIANT-4 trial. There were 40 patients with rectal NET included in a subgroup analysis, which resulted in prolonged PFS with everolimus compared to placebo [36]. ENETS recommend that everolimus may be used as second-line therapy in metastatic rectal NET who have progressed. It may also be used as first-line systemic therapy in patients who do not demonstrate somatostatin receptor expression on somatostatin receptor based functional imaging, and are therefore unsuitable for SSAs.

#### Tyrosine Kinase Inhibitors (TKIs)

Sunitinib has been shown to prolong PFS in pancreatic NENs but there is no evidence in extra-pancreatic NENs and no published phase III trials of sunitinib in rectal NETs. Other TKIs such as levantinib, surufatinib and sorafenib have been proposed in phase II trials. The SANET-ep phase III trial of surufatinib included 53 patients with rectal NET [37]. The PFS was longer for the surufatinib group compared to placebo, however no subgroup analysis on rectal NETs was published. Similarly, the AXINET phase III trial of axitinib + SSA included 16 rectal NETs and showed improved PFS for the whole study population in the axitinib group [38]. The ongoing CABINET phase III trial (NCT03375320) has recently published interim analysis which demonstrates that it has met its primary endpoint of prolonged PFS in the cabozantinib group compared to placebo [39]. This trial included patients that had had progressive disease after prior systemic therapy in advanced pancreatic and extra-pancreatic NETs, although as yet it is unreported whether rectal NETs were included.

More data is needed to determine the role of TKIs in progressive rectal NETs. ENETS recommend TKIs may be used after failure of better-established treatment options (everolimus, PRRT, locoregional treatments) ideally within a clinical trial [11].

#### Chemotherapy

Chemotherapy is rarely indicated in well-differentiated rectal NETs, but is used more commonly in poorly differentiated NECs. The BETTER trial included 7 patients with colorectal NETs. There was an overall disease control rate of 88% for bevacizumab and capecitabine [40]. In NECs, cisplatin or carboplatin in combination with etoposide is considered first line therapy [41].

##### PRRT

^177^Lutetium-Dotatate peptide receptor radionuclide therapy (PRRT) for the treatment of well-differentiated midgut somatostatin receptor positive NETs have been associated with a significant improvement in PFS. The major trial (NETTER-1) did not include rectal NENs [42]. A retrospective study of 27 rectal NEN patients who were treated with PRRT showed that 70% of patient had a partial response and 26% had stable disease [43]. Treatment was well tolerated with minimal toxicity.

#### Potential New Therapies

There are currently few targeted therapy treatment options for rectal NETs. Two recently published studies performed whole exome sequencing on rectal tumour cells looking for potentially targetable mutations. One study found 72.2% of tumours carried at least one potentially targetable gene mutation [44]. There was a high frequency variation in the PI3K-AKT signaling pathway which was proposed to suggest a high sensitivity to the mTOR inhibitor everolimus. BRAF mutations were also common. There were several gene alterations which were associated with worse overall survival which could be potentially targeted in the future. Another study of 38 tumours found that poorly differentiated rectal NETs that were more likely to metastasize tended to have mutations in the Wnt, MAPK and PI3K/AKT signaling pathways which could be potential targets in this group [45]. These studies were both single-centre studies which may affect the applicability of these results.

A systematic review of genetic mutations in rectal NETs looked for potentially targetable mutations [46]. The most frequently mutated genes were APC, TP53, KRAS, BRAF, RB1, CDKN2A and PTEN. BRAF mutations were found in 7.1 to 33.3% of rectal NETs. Patients with these mutations may be amenable to combination BRAF and MEK inhibition when they have progressed despite chemotherapy. More understanding of the genetic alterations in rectal NETs can aid with risk stratification and predicting prognosis, and open the possibility of individualized management.

## Conclusion

Rectal NETs require a multidisciplinary team approach to increase the chance of complete resection and therefore reduce the risk of recurrence. Resection technique depends on tumour size, presence of muscularis propria or lymphovascular invasion and presence of distal metastases. Endoscopic resection can be considered for lesions < 20 mm with no nodal involvement, but the resection technique depends on size and muscle layer involvement. Follow up will depend on tumour size, grade and the presence of lymphovascular and nodal involvement, and whether the tumour was completely resected. Many of the published studies of rectal NETs are retrospective and single-centre. Large multicenter cohort studies to better determine management options would be helpful to guide management.

## Key References

- Fine C, Roquin G, Terrebonne E, Lecomte T, Coriat R, Do Cao C, et al. Endoscopic management of 345 small rectal neuroendocrine tumours: A national study from the French group of endocrine tumours (GTE). United European Gastroenterology Journal. 2019;7(8):1102–12.
  - ○ This reference is of importance because it illustrates that in Europe high rates of incomplete resection can occur due to insufficient recognition of neuroendocrine neoplasms at colonoscopy. Training endoscopists to better recognise these lesions is of high importance.
- Rinke A, Ambrosini V, Dromain C, Garcia-Carbonero R, Haji A, Koumarianou A, et al. European Neuroendocrine Tumor Society (ENETS) 2023 guidance paper for colorectal neuroendocrine tumours. J Neuroendocrinol. 2023;35(6):e13309.
  - ○ This reference is of importance because it provides an evidence based guideline for management of colorectal neuroendocrine neoplasms.
- Choi JS, Kim MJ, Shin R, Park JW, Heo SC, Jeong SY, et al. Risk Factor Analysis of Lymph Node Metastasis for Rectal Neuroendocrine Tumors: Who Needs a Radical Resection in Rectal Neuroendocrine Tumors Sized 1–2 cm? Ann Surg Oncol. 2024.
  - ○ This reference is of importance as previously there has been little data on selecting resection technique in rectal NETs 10–20 mm in size. They suggest that tumours in this size category who are grade 2 or 3 should undergo surgical resection rather than endoscopic due the higher risk of lymphovascular invasion.
- Cheminel L, Lupu A, Wallenhorst T, Lepilliez V, Leblanc S, Albouys J, et al. Systematic Resection of the Visible Scar After Incomplete Endoscopic Resection of Rectal Neuroendocrine Tumors. Am J Gastroenterol. 2024;119(2):378–81.
  - ○ This reference is of importance because it demonstrated that in small rectal NETs < 10 mm who have had an incomplete resection, there was residual disease in 43% of scars. A second endoscopic procedure lead to complete resection in 75.6% of these cases, who were then able to be discharged from further surveillance.

## Data Availability

No datasets were generated or analysed during the current study.
